# Supplementary material for: Validation of a Deep Learning Model for Detecting Chest Pathologies from Digital Chest Radiographs
Source: Diagnostics (Basel). 2023 Feb 2;13(3):557. doi: 10.3390/diagnostics13030557 (PMC9914339; doi:10.3390/diagnostics13030557)
Supplement: Supplementary file 1 [file diagnostics-13-00557-s001.zip › diagnostics-2133982-supplementary.pdf]

Validation of a deep learning model for detecting chest pathologies from digital chest radiographs

SUPPLEMENTARY DATA

		AUROC [95% CI]		Sensitivity [95% CI]		Specificity [95% CI]	
**Hospital**	**Reader**	**Unaided Session**	**Aided Session**	**Unaided Session**	**Aided Session**	**Unaided Session**	**Aided Session**
A	R1	0.843 [0.812,0.874]	0.861 [0.831, 0.888]	0.723 [0.662,0.785]	0.821 [0.767,0.871]	0.963 [0.949, 0.975]	0.9003 [0.878, 0.921]
	R2	0.881 [0.854,0.908]	0.901 [0.878, 0.923]	0.802 [0.749, 0.855]	0.915 [0.876, 0.950]	0.959 [0.945, 0.973]	0.888 [0.864, 0.909]
	R3	0.835 [0.806,0.867]	0.869 [0.839,0.896]	0.787 [0.732, 0.843]	0.815 [0.759, 0.866]	0.884 [0.859, 0.906]	0.923 [0.905, 0.941]
B	R1	0.884 [0.819,0.942]	0.885 [0.818,0.944]	0.7914 [0.667, 0.903]	0.816 [0.688,0.927]	0.977 [0.948, 1.000]	0.953 [0.912, 0.991]
	R2	0.881 [0.813,0.942]	0.923 [0.870, 0.969]	0.798 [0.667, 0.915]	0.902 [0.806, 0.977]	0.964 [0.926, 0.992]	0.945 [0.902, 0.983]
	R3	0.600 [0.532,0.676]	0.872 [0.807, 0.928]	0.258 [0.128, 0.395]	0.853 [0.738, 0.948]	0.943 [0.901, 0.982]	0.892 [0.835, 0.945]
Entire dataset	R1	0.849 [0.821,0.876]	0.864 [0.838, 0.889]	0.734 [0.678, 0.788]	0.820 [0.771, 0.866]	0.965 [0.952, 0.976]	0.908 [0.888, 0.926]
	R2	0.881 [0.855,0.906]	0.904 [0.883, 0.923]	0.801 [0.751, 0.849]	0.913 [0.877, 0.946]	0.960 [0.947, 0.973]	0.895 [0.875, 0.915]
	R3	0.796 [0.767,0.826]	0.870 [0.844, 0.895]	0.701 [0.647, 0.757]	0.821 [0.771, 0.867]	0.892 [0.870, 0.912]	0.919 [0.901, 0.937]

Supplementary Table S1: Aggregate performance of the human readers in session 1 (Unaided Session) and session 2 (Aided Session) across all categories in external validation tests.

Reader	Average time ± SD to read one scan (in seconds)		p-value
	Unaided session	Aided session	
R1	9.99 ± 15.07	6.16 ± 4.4	<0.01
R2	11.18 ± 12.91	9.04 ± 3.5	0.044
R3	19.13 ± 37.67	16.63 ± 57.51	<0.01
Average (R1, R2, R3)	13.43±24.92	10.61±33.66	<0.01

Supplementary Table S2: Analysis of chest radiograph interpretation time by readers during unaided and aided reading sessions.
